# Artificial Intelligence-Guided Discovery of Covalent Organic Frameworks for Next-Generation Polyfluoroalkyl Substances Removal

**DOI:** 10.1021/acsami.6c05236

**Published:** 2026-06-25

**Authors:** Asmaa Jrad, Ali Trabolsi

**Affiliations:** † Chemistry Program, Science Division, New York University Abu Dhabi, 129188 Abu Dhabi, UAE; ‡ NYUAD Water Research Center, New York University Abu Dhabi, 129188 Abu Dhabi, UAE

**Keywords:** PFAS remediation, covalent organic frameworks, artificial intelligence, machine learning, water treatment, adsorption, selective polishing, porous materials

## Abstract

The pervasive threat of per- and polyfluoroalkyl substances (PFAS) necessitates water treatment solutions capable of selective, trace-level removal within chemically complex, high-flux environments. Covalent organic frameworks (COFs) offer an unparalleled platform for such precision remediation through rational pore-surface engineering. However, industrial translation is currently constrained by a multiobjective optimization bottleneck, where adsorption performance must be balanced against manufacturability, end-of-use strategy, and economic viability. In this perspective, we bridge the gap between idealized laboratory studies and industrial requirements, positioning COFs not as bulk sorbent replacements, but as high-fidelity selective polishing layers within hybrid treatment trains. We argue that artificial intelligence (AI) and data-driven methodologies provide a critical framework for navigating this complex landscape, serving as an integrative layer to accelerate candidate screening, refine structure-performance descriptors through characterization-informed feedback, and enable process-aware optimization. Finally, we highlight the current absence of AI-guided discovery strategies explicitly tailored for COF-enabled PFAS removal in realistic aqueous matrices and propose a pragmatic roadmap toward interoperable data sets and closed-loop workflows linking molecular design to manufacturable architectures and real-water performance.

## Graphical abstract

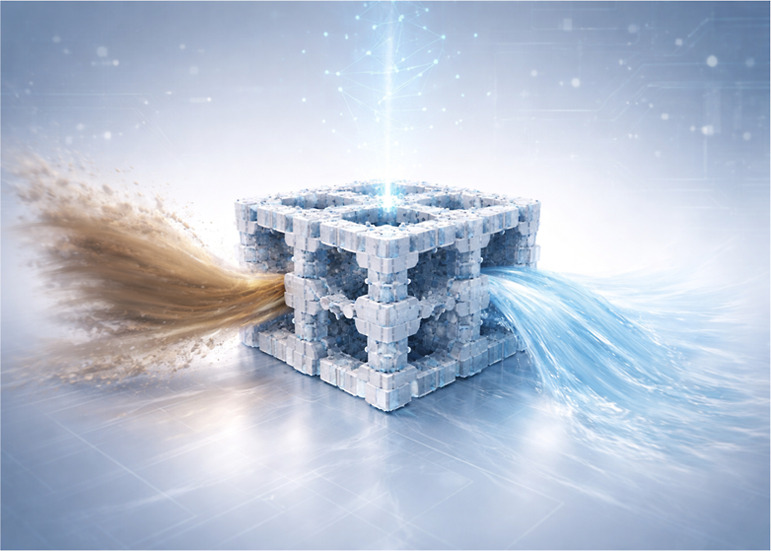

## 1 Introduction

Per- and polyfluoroalkyl substances (PFAS) contamination has emerged as one of the most significant environmental and economic challenges of the 21st century.^1^ Often termed “forever chemicals” due to the strength of the carbon–fluorine bond, these compounds lead to chronic bioaccumulation.^2^ This environmental crisis has triggered a massive global market response. Recent estimates suggest the global PFAS remediation and sensing market is projected to exceed $2.5 billion USD by 2030, driven by stringent new regulations like the 2026 EU Drinking Water Directive and the US EPA’s maximum contaminant levels (MCLs).^3^

This “regulatory hammer” has created an immediate industrial demand for innovative materials capable of high-efficiency removal, particularly in complex industrial wastewater and municipal drinking water systems.^4^ However, existing treatment infrastructure, primarily reliant on granular activated carbon (GAC) and ion-exchange resins, faces important limitations, including nonspecific adsorption, reduced efficiency under competitive conditions such as natural organic matter, and challenges associated with regeneration and long-term performance.^5–7^ This highlights a broader limitation of traditional adsorbents, namely, the stochastic pore architectures and limited chemical tunability. There is a clear commercialization gap; the market needs materials that are not only high-performing but also scalable and manufacturable.

Crystalline porous materials offer a fundamentally different design paradigm compared to conventional materials, enabling the rational placement of functional groups and precise control of pore environments.^8^ Within this landscape, covalent organic frameworks (COFs) have surfaced as a transformative platform for precision remediation. Unlike traditional adsorbents, COFs offer a “designer” approach, where crystalline structures allow for the predetermined placement of functional groups to exploit both hydrophobic and electrostatic interactions for efficient PFAS remediation.^9^ Yet, the chemical space for these materials is virtually infinite, and the traditional “trial-and-error” synthesis cycle is too slow to meet the expanding market demand and the urgent timeline of environmental protection agencies.^10^

This perspective argues that the next generation of PFAS remediation will be defined by the integration of AI-guided frameworks and process simulation. By utilizing machine learning to navigate the complex structure-performance landscape, we can accelerate the discovery-to-market pipeline, transforming COFs from lab-scale curiosities into the scalable, industrial-grade solutions the global water sector is currently demanding. In the sections that follow, we first frame COF development for PFAS removal as a multiobjective commercialization problem (performance, synthesis, economic viability, end-of-use strategy), then identify the key bottlenecks that emerge specifically in real-water and practical applications. We next outline where AI can credibly accelerate progress, ranging from structure generation and screening, to characterization-informed model refinement, to process-aware optimization for batch and continuous-flow operation. Finally, we propose a pragmatic closed-loop workflow that links digital design to experimentally validated performance in realistic matrices and scalable architectures.

## 2 Current Landscape and the Multi-Objective Challenge of Covalent Organic Frameworks Design

To date, the development of COFs for PFAS remediation has primarily focused on maximizing adsorption capacity and kinetics.^9^ By utilizing cationic frameworks^8,11^ or tuning pore surfaces with hydrophobic/fluorophilic moieties,^12–16^ researchers have achieved remarkable parts-per-billion (ppb) to parts-per-trillion (ppt) removal efficiencies.^8,9^ However, no universal terminal PFAS concentration can be assigned to COFs as a class, because reported end points depend on the COF structure, PFAS identity, initial concentration, adsorbent dose, contact time, water matrix, analytical detection limit, and whether testing is performed in batch or flow-through mode. Therefore, compliance with emission or drinking-water standards must be assessed case-by-case under standardized, matrix-relevant conditions rather than inferred from removal percentage alone. Representative examples illustrate that the unique value of COFs for PFAS removal arises from the tunable spatial arrangement of pore size, charge, hydrophobicity, and accessible binding sites. Cationic COFs, including guanidinium- or ionic-site-containing frameworks, exploit electrostatic attraction with anionic PFAS headgroups, while hydrophobic/fluorophilic, β-cyclodextrin-derived, and nitrogen-rich frameworks introduce complementary interactions with the fluorinated tail or provide confinement within tailored pores. Across these systems, PFAS uptake is generally governed by combinations of electrostatic attraction, hydrophobic association, fluorophilic interactions, host–guest inclusion, pore confinement, and binding-site accessibility. These examples support the central viewpoint of this perspective: future COF development should move beyond reporting isolated adsorption performance and instead connect molecular design motifs to deployment-relevant metrics such as selectivity, matrix tolerance, kinetics, processability, and cost. Thus, as the field transitions from laboratory discovery to practical application, the criteria for a “successful” COF expand into a multidimensional optimization problem.

Foremost is the requirement of performance, encompassing high equilibrium removal capacity, rapid kinetics, and selectivity under realistic flow and concentration regimes. Yet high-performing frameworks must also meet the demands of manufacturability, where synthetic simplicity and adherence to green chemistry principles become critical.^17^ Industrial translation necessitates high-yield, scalable synthesis protocols that minimize synthetic complexity, avoid expensive catalysts, and reduce reliance on environmentally burdensome solvents^18^ In parallel, the end-of-use strategy must be considered in a deployment-specific manner. For industrial or nonpotable treatment systems, regenerability may be critical to reduce material consumption and operating costs, requiring a balance between strong PFAS binding and nondestructive desorption. However, in ultrapolishing applications for drinking water, where COFs would target residual PFAS at very low concentrations, single-use cartridges or replaceable polishing layers may be more realistic than repeated in situ regeneration, particularly if organic regenerants pose risks of residual leaching. Therefore, regeneration studies should be designed with deployment context in mind, prioritizing benign, easily removable, and industrially manageable regenerants such as ethanol-based systems, aqueous salt/solvent mixtures, pH-triggered desorption, or mild electrochemical/thermal approaches, accompanied by rigorous postregeneration rinsing and leachate analysis.^19^ Finally, economic viability imposes an overarching constraint, as material costs, processing requirements, and deployment feasibility ultimately govern large-scale adoption.^20^ COF cost is structure- and process-dependent, varying with linker availability, synthetic route, solvent demand, yield, purification requirements, postsynthetic modification, and final form factor. Cost should therefore be treated as a design variable rather than a fixed property of COFs as a class.

Within traditional experimental paradigms, optimization along one dimension frequently incurs penalties along others. Frameworks engineered for exceptional adsorption performance may require synthetically demanding routes, while materials designed for facile synthesis and processing may sacrifice adsorption efficiency or selectivity.

This multiobjective reality fundamentally reframes COF development as a Pareto optimization problem, in which adsorption performance, synthetic feasibility, material synthesis cost, and end-of-use strategy must be balanced simultaneously. Under this lens, the challenge is no longer the identification of a material that merely “works,” but rather the discovery of frameworks that occupy the optimal intersection of these competing constraints. This is where the true design horizon emerges, since the chemical space of COFs is too vast to be navigated through human intuition and sequential experimentation alone.^21^ Consequently, data-driven and AI-guided discovery strategies become not just advantageous but necessary. Such approaches could help prioritize hypothetical COF structures for experimental validation by identifying candidates predicted to better balance performance, manufacturability, end-of-use strategy, and economic viability, while recognizing that PFAS-specific validation under realistic conditions remains largely unexplored.^22^ Figure 1 schematically illustrates this shift from trial-and-error COF discovery toward AI-guided multiobjective design. In the radar plot, the performance-focused profile of a conventional COF is contrasted with a more balanced AI-optimized design space that simultaneously considers performance, manufacturability, end-of-use strategy, and economic viability. The plot is intended as a qualitative illustration of these design trade-offs and does not represent quantitatively normalized scores.

**Figure 1:**
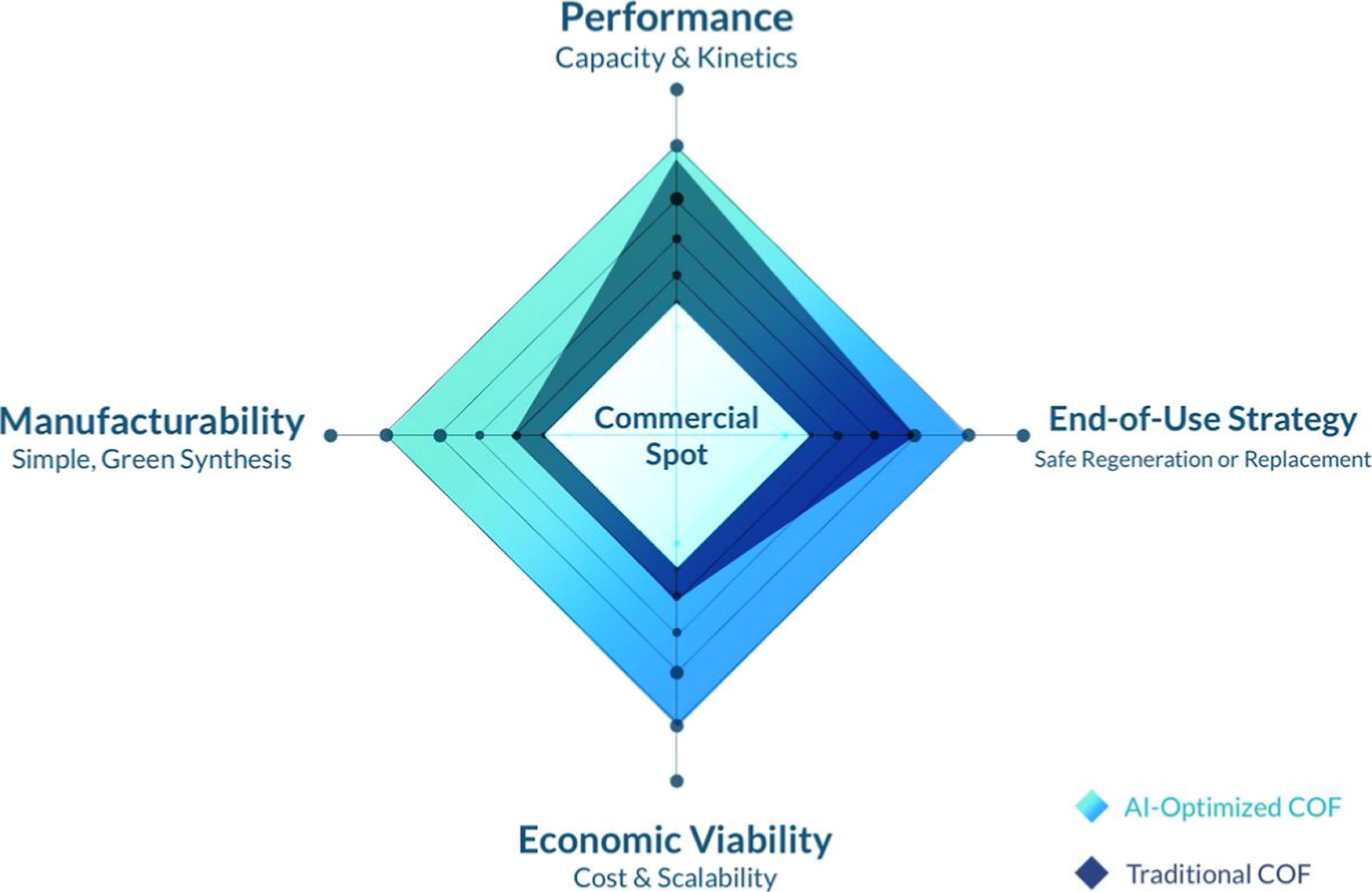
Schematic multiobjective design space for COF deployment in PFAS remediation. The radar plot conceptually illustrates the transition from performance-dominated COF development toward AI-guided optimization that balances adsorption performance, manufacturability, end-of-use strategy, and economic viability. The plotted profiles are illustrative and do not represent normalized quantitative scores.

## 3 Practical Bottlenecks: From Idealized Systems to Real Water

While the multiobjective constraints discussed above define the challenges of COF design, the practical deployment of these materials introduces an additional and equally critical dimension. The gap between laboratory demonstrations and industrial water treatment requirements remains substantial, reflecting not only differences in operating environments but also fundamental differences in technological roles.

Much of the existing academic literature implicitly treats COFs as standalone solutions, often evaluating their performance as if they were intended to function as the sole treatment medium for an entire aqueous matrix.^23^ In practice, however, industrial-scale water treatment systems rely on cost-effective bulk technologies such as granular activated carbon (GAC) and ion-exchange (IX) resins, which offer robust performance across a wide range of contaminants.^24^ Given the economic and operational advantages of these conventional adsorbents, COFs are unlikely to serve as wholesale replacements for these established materials. Instead, their immediate technological relevance lies in functioning as precision polishing layers within hybrid treatment trains, specifically targeting PFAS species that challenge conventional adsorbents. This repositioning fundamentally reframes the application landscape. Rather than maximizing universal adsorption capacity, COFs must operate as selective molecular scavengers embedded within chemically complex and highly competitive aqueous environments. Although GAC and IX resins remain effective and economically favorable for bulk PFAS removal, increasingly stringent concentration limits, residual PFAS breakthrough, short-chain PFAS persistence, competitive matrix effects, and regeneration-related operational burdens continue to motivate the development of more selective and efficient adsorbents. This need is further reflected by dedicated funding initiatives from agencies such as the U.S. EPA,^25^ NSF,^26^ and SERDP/ESTCP,^27^ which have supported research into improved PFAS treatment technologies, including novel sorbents and treatment-train approaches. While COFs have not yet been commercialized or validated at pilot scale specifically for PFAS removal, the commercialization of β-cyclodextrin-derived DEXSORB materials by CycloPure illustrates that molecularly engineered sorbents can be tailored for PFAS capture in real water matrices and translated toward commercial use when they address a clearly defined treatment niche.^28^ More broadly, the emergence of commercial PFAS-specific adsorbent media and filtration systems further indicates that the market is moving toward targeted molecular capture strategies rather than relying exclusively on conventional bulk adsorption.^29^ In this context, COFs should be evaluated not as stand-alone replacements for conventional adsorbents, but as tunable precision layers that could enhance PFAS removal within multistage treatment architectures.

A second bottleneck arises from the fact that most reported studies emphasize single-solute PFAS removal in simplified aqueous systems, providing limited insight into behavior under realistic competitive conditions.^30^ The absence of consistent benchmarks for “dirty water” performance severely constrains predictive design, rendering intuition-driven materials selection increasingly unreliable. Real-world water matrices are dominated by natural organic matter (NOM), dissolved salts, and fluctuating physicochemical conditions, where PFAS concentrations often reside at trace levels (ppt to ppb).^31^ Under such conditions, selectivity becomes the defining bottleneck. Trace contaminants must be captured amidst orders-of-magnitude higher concentrations of competing species, demanding exceptionally precise pore-surface engineering. Yet predicting which structural motifs will maintain this molecular discrimination in the presence of NOM and competing ions remains inherently difficult.

Compounding these difficulties are form-factor considerations. Laboratory-scale COFs are frequently synthesized as fine powders, a morphology incompatible with high-throughput continuous-flow systems due to pressure-drop constraints, mechanical instability, and handling limitations.^9^ Translation into deployable architectures, such as membranes, beads, coatings, or composites, introduces additional complexity, as structural accessibility, diffusion pathways, and adsorption behavior may deviate significantly from powder-based metrics.^32^ Maintaining performance while achieving mechanical robustness, therefore, represents a nontrivial materials engineering challenge. Thus, the primary processing difficulty is not only identifying high-affinity COF chemistries but preserving active-site accessibility and adsorption kinetics after converting powders into hydraulically stable, scalable formats.

At present, no COF class can be described as “widely used” for PFAS removal in practical water-treatment systems; the field remains largely at the laboratory scale. Among reported systems, cationic and hydrophobic/fluorophilic frameworks currently provide some of the clearest mechanistic rationale for high PFAS affinity, but the “best” material depends on the target PFAS, matrix composition, concentration regime, adsorbent form, and performance metric used for comparison.

Collectively, these constraints redefine the central bottleneck. The challenge is no longer our ability to synthesize new COFs, but rather our ability to predict which frameworks will function reliably within realistic treatment architectures and hybrid infrastructures. Exhaustive trial-and-error exploration of this chemical and operational space is economically and temporally impractical, underscoring the need for integrative, data-driven strategies.

This disconnect between idealized laboratory evaluation and practical deployment is summarized in Figure 2. The scheme contrasts conventional single-solute batch testing, which is useful for mechanistic screening, with the more complex reality of PFAS remediation, where COFs must be evaluated in competitive water matrices, after bulk adsorption stages, and within deployable flow-through architectures.

**Figure 2:**
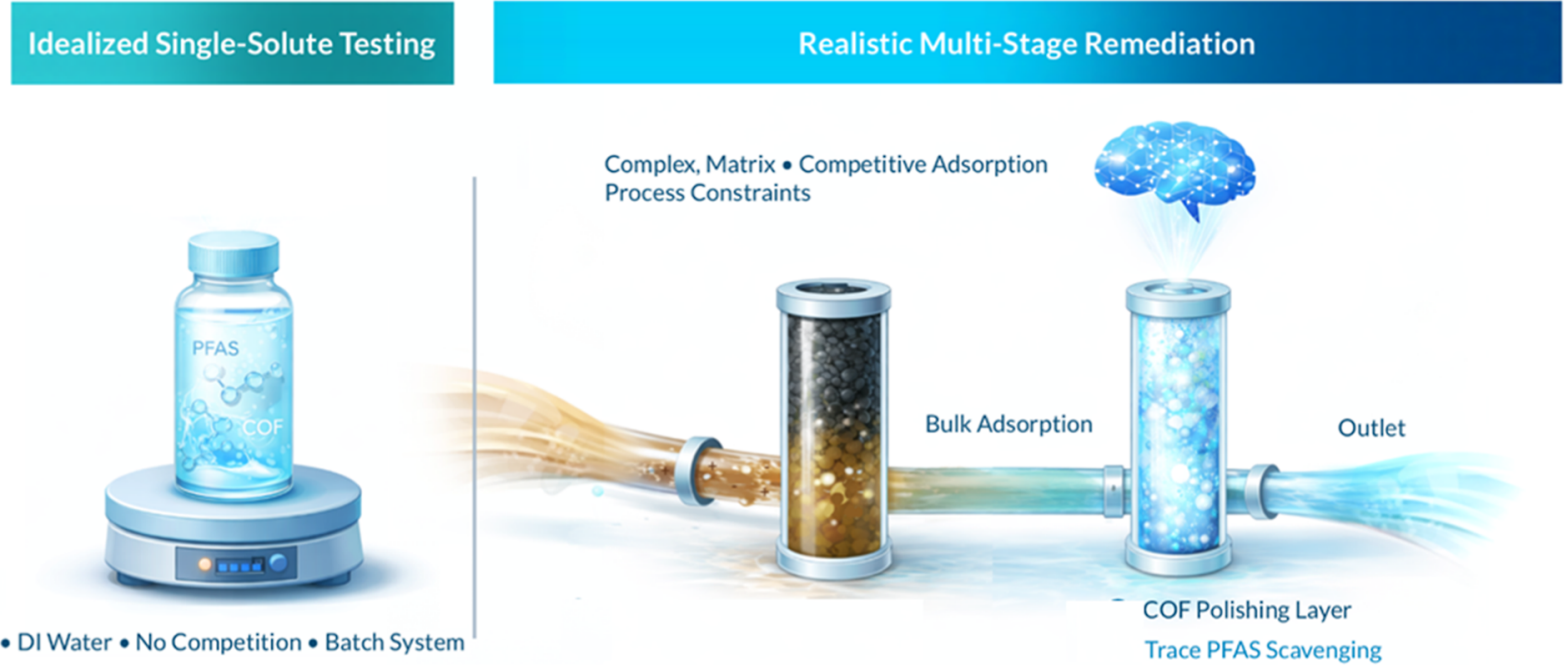
Conceptual illustration of the disconnect between idealized laboratory evaluation of COFs and multiconstraint real-world PFAS remediation. Comparison between single-solute batch experiments and multistage treatment systems where COFs function as selective polishing layers after bulk adsorption.

## 4 The Role of Artificial Intelligence in Covalent Organic Framework Design and Deployment

The growing complexity of COF design and deployment challenges has catalyzed increasing interest in artificial intelligence and data-driven methodologies within the COF research community. While AI has historically been more extensively explored in adjacent porous materials domains, recent studies demonstrate that COFs are increasingly being integrated into computational and machine-learning-assisted discovery frameworks. These developments provide an important foundation for evaluating how AI can credibly accelerate COF innovation for PFAS remediation.

### 4.1 Artificial Intelligence-Assisted Materials Discovery, Synthesis Guidance, and Quick Characterization

One of the earliest and most natural entry points for AI in COF research lies in materials discovery and high-throughput screening.^33^ The modular nature of COFs, defined by the combinatorial assembly of organic linkers and nodes, creates a vast chemical design space that rapidly exceeds the limits of intuition-driven exploration. Recent computational studies have addressed this challenge through the construction of large hypothetical COF libraries, enabling systematic evaluation of structure–property relationships.^34,35^

Machine-learning (ML) models trained on structural descriptors have been employed to predict adsorption-relevant properties, including surface area, pore size distributions, and guest–framework interaction trends.^35^ Such approaches significantly reduce computational burden compared to exhaustive atomistic simulations, allowing rapid prioritization of candidate structures. Importantly, these strategies do not replace experimental validation but instead function as a guiding mechanism, narrowing the search space toward synthetically and functionally promising frameworks.

Beyond structural screening, emerging studies highlight the integration of AI within experimental synthesis workflows. Recent reports demonstrate human–AI collaborative strategies in which ML models guide the selection of building blocks, reaction conditions, or synthetic routes, followed by experimental validation.^36^ Such approaches are especially valuable in COF chemistry, where synthetic success depends on delicate balances among precursor reactivity, reversibility, crystallization kinetics, and defect formation. AI-assisted optimization frameworks, including Bayesian optimization and active learning strategies, can support synthesis development by guiding the selection of reaction conditions that are more likely to yield crystalline, reproducible, and experimentally viable COFs.^37,38^ These studies illustrate that AI already functions as a practical decision-support tool within COF laboratories. Rather than representing speculative future capabilities, AI-driven synthesis optimization constitutes an evolving methodological layer capable of reducing experimental trial-and-error, resource consumption, and development timelines.

A less explored but increasingly important dimension of AI-assisted COF research lies in characterization and structural validation. COF performance is highly sensitive to crystallinity, defect landscapes, pore accessibility, and functional group distribution. Experimental characterization techniques generate rich yet complex data sets that are often interpreted qualitatively or semiquantitatively. Recent advances demonstrate the application of computational intelligence and data-driven approaches to COF structure determination, including electron crystallography-assisted analysis and automated diffraction interpretation.^39^ These developments highlight the potential for AI to assist not only in materials prediction but also in extracting actionable insights from experimental measurements.

More broadly, ML methodologies offer opportunities to correlate characterization-derived features, such as spectroscopic signatures, porosity metrics, or regeneration-induced structural deviations, with adsorption performance.^40^ In this framework, characterization data evolve from purely diagnostic outputs into active inputs for descriptor refinement and predictive modeling. Such strategies are particularly relevant for understanding regeneration stability, failure modes, and performance variability across form factors, addressing key bottlenecks in COF deployment.

### 4.2 Artificial Intelligence as a Surrogate for Computationally Intensive Simulations

Atomistic simulations, including molecular dynamics (MD) and density functional theory (DFT), remain indispensable for probing guest-framework interactions. However, their computational cost limits applicability across large hypothetical materials spaces. AI-based surrogate models provide an increasingly viable solution to this limitation.^41^

Studies within COF and porous materials research have demonstrated that ML models trained on targeted simulation outputs can approximate binding energies, diffusion coefficients, and interaction trends at significantly reduced computational expense.^42^ Hybrid workflows combining selective high-fidelity simulations with ML interpolation enable broader exploration of chemical spaces while preserving physical interpretability.

Crucially, surrogate modeling should be viewed as an acceleration strategy rather than a replacement for physics-based methods. When integrated judiciously, AI surrogates expand computational reach while maintaining mechanistic grounding.

### 4.3 Artificial Intelligence-Aware Process Modeling and Deployment Optimization

The performance of COFs cannot be decoupled from process-level considerations. Recent computational frameworks emphasize the integration of materials descriptors with system-level modeling, enabling prediction of adsorption behavior within batch systems, columns, and hybrid treatment architectures.^43^

Digital twin methodologies and ML-assisted modeling approaches offer opportunities to simulate breakthrough behavior, optimize layer thicknesses, and evaluate operational constraints prior to pilot-scale implementation.^44,45^ These strategies align closely with the emerging role of COFs as precision polishing layers rather than standalone treatment media.

## 5 Limitations, Gaps, and the Path Toward Autonomous Discovery

Despite the rapid expansion of AI-assisted methodologies across COF research, their application to PFAS remediation remains notably underdeveloped. While machine learning and data-driven frameworks have begun to influence COF discovery, synthesis optimization, and structural analysis, no systematic AI-guided design strategies have yet been established specifically for COFs targeting PFAS removal in realistic aqueous environments. This disconnect highlights a broader methodological gap between advances in computational materials science and their translation into environmental remediation challenges.

Several factors contribute to this limitation. Foremost is the scarcity of high-quality, standardized data sets relevant to PFAS adsorption. Existing experimental studies often emphasize single-solute systems under idealized conditions, providing limited insight into competitive adsorption behavior, regeneration stability, and long-term structural robustness.^9^ The absence of consistent reporting standards further complicates data aggregation, as variations in experimental protocols, performance metrics, and characterization practices introduce significant descriptor noise.

Additionally, negative experimental outcomes, such as failed syntheses, poor adsorption performance, or regeneration-induced degradation, remain largely underreported, despite their statistical importance for robust ML model development.^46^ Without such data, AI frameworks risk inheriting bias toward successful systems while lacking predictive reliability across broader chemical spaces.

A second challenge lies in descriptor transferability. COF performance emerges from complex interdependencies among pore architecture, functional group distribution, crystallinity, defect landscapes, and operating conditions. AI models trained on limited or idealized data sets may exhibit reduced predictive accuracy when extrapolated to multicomponent aqueous matrices or alternative form factors. This limitation underscores the necessity of integrative workflows capable of linking digital predictions with experimentally validated behavior.

Addressing these constraints requires a paradigm shift toward closed-loop, data-centric discovery frameworks. We envision the development of interoperable PFAS–COF databases that integrate structural descriptors, adsorption performance, regeneration stability, characterization outputs, and negative experimental data. Such platforms would enable active learning strategies, where AI models iteratively refine predictive accuracy through continuous feedback from experimental validation.

To avoid training AI models toward laboratory artifacts rather than practical performance, PFAS–COF data sets should distinguish explicitly between testing contexts rather than aggregating all adsorption data into a single undifferentiated label. Single-solute batch experiments in deionized water provide useful mechanistic information, but they primarily reflect intrinsic adsorption capacity under idealized conditions. In contrast, real-world polishing applications require metrics related to selectivity, residual PFAS concentration, breakthrough behavior, matrix tolerance, and performance retention in the presence of natural organic matter, dissolved ions, and competing contaminants. Therefore, future data sets should include metadata describing water matrix composition, PFAS mixture complexity, concentration regime, adsorbent form factor, contact time, flow conditions, and treatment-train position.

Actionable AI-guided discovery will also require physically meaningful descriptor selection. Relevant descriptors should span (i) framework structure, including pore size, pore volume, interlayer spacing, topology, and defect density, charge density, cationic site density, hydrophobicity, fluorophilic group density, and electrostatic potential; (ii) PFAS identity, including chain length, headgroup chemistry, molecular volume, and charge distribution; and (iii) process conditions, including pH, ionic strength, natural organic matter concentration, empty-bed contact time, and flow rate. Quantifying these descriptors would enable models to move beyond simple capacity prediction toward task-specific outputs such as selectivity factors, matrix tolerance indices, breakthrough volumes, residual PFAS concentration, and performance retention factors.

Given the limited number of COF studies conducted under realistic aqueous matrices, near-term AI models should also draw information from adjacent PFAS adsorption literature involving related porous and polymeric adsorbents, including MOFs, POPs, β-cyclodextrin-based polymers, GAC, and IX resins. These data sets are particularly valuable when the same material has been tested under both idealized and realistic conditions, allowing models to learn how adsorption capacity, kinetics, selectivity, and breakthrough behavior change as a function of matrix complexity. Rather than treating DI-water and real-water results as equivalent, AI workflows could estimate a matrix correction or performance retention factor that relates idealized adsorption behavior to expected performance under competitive conditions. Such factors would not be intended as exact substitutes for experiments, but as practical ranking tools for comparing candidate COFs and prioritizing those most likely to retain performance in real water.

Molecular simulations can further support this translation by providing mechanistic insight into matrix effects that are difficult to isolate experimentally. Molecular dynamics simulations in the presence of competing ions, natural organic matter surrogates, and mixed PFAS compositions could help quantify how pore accessibility, diffusion, hydration, and binding-site competition influence adsorption performance. These simulation-derived descriptors could be incorporated into multifidelity models, where single-solute adsorption data serve as low-fidelity mechanistic inputs, real-water and flow-through experiments serve as high-fidelity deployment labels, and MD-derived matrix factors provide an intermediate bridge between the two. Such a framework would help prevent AI models from overvaluing materials that perform well only under idealized conditions and would instead guide discovery toward COFs optimized for realistic polishing-layer function.

From an algorithmic perspective, the limited and heterogeneous nature of PFAS–COF data suggests that physics-informed, transfer-learning, and multifidelity learning strategies may be more appropriate than purely black-box models. Active learning and Bayesian optimization could then be used to prioritize experiments that maximally reduce model uncertainty, particularly in poorly sampled regions of chemical and operational space. In practice, the objective would not be to predict real-water performance with absolute certainty, but to generate experimentally grounded rankings and uncertainty estimates that guide which COFs should be synthesized, modified, or tested next under process-relevant conditions.

Within this framework, AI-driven simulations and surrogate models could prioritize candidate structures optimized not only for adsorption performance but also for manufacturability, regenerability, and economic feasibility. Crucially, experimental laboratories remain central to this cycle, functioning as validation engines that anchor computational predictions in physical reality.

This transition fundamentally redefines the role of the researcher. Rather than operating solely as a materials generator, the scientist increasingly functions as a systems architect, managing the dynamic exchange of information between digital design environments and experimental platforms. In the context of PFAS remediation, such integrative discovery ecosystems offer a credible pathway for accelerating the identification of COFs capable of operating within realistic treatment architectures.

The proposed workflow is illustrated in Figure 3, which links AI-guided COF selection, molecular simulation, synthesis, characterization, and real-water testing within an iterative feedback loop. The lower portion of the scheme highlights how optimized COF materials could then be translated into practical PFAS-removal formats, ranging from point-of-use filters to larger treatment-train configurations.

**Figure 3:**
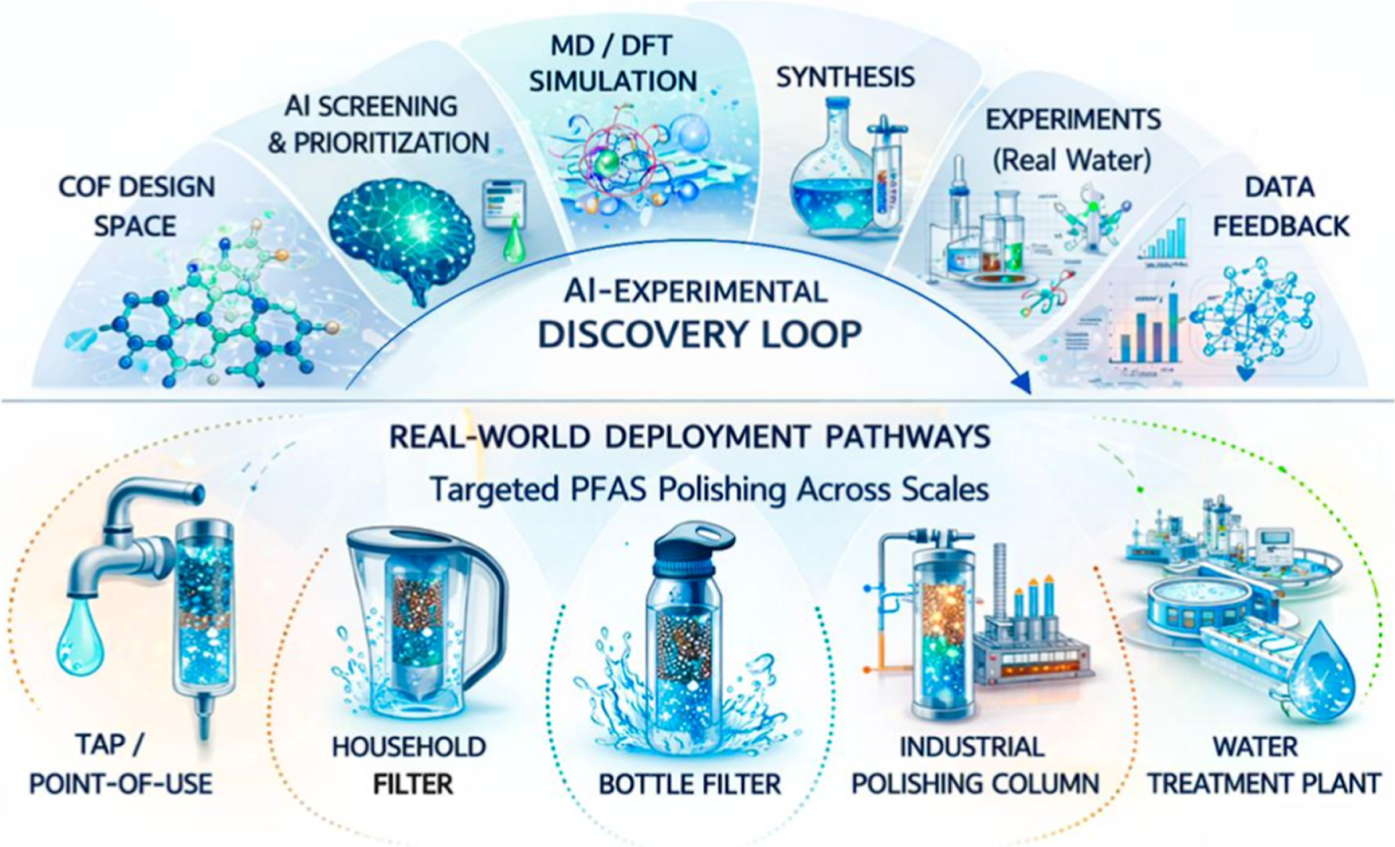
AI-experimental discovery loop accelerating COF development from molecular design to real-world PFAS remediation across deployment scales.

## 6 Vision and Future Outlook: Toward Autonomous Remediation

The true horizon for porous materials extends beyond the incremental discovery of higher-performing frameworks. It lies in the emergence of integrated, adaptive, and ultimately autonomous remediation ecosystems. In this evolving paradigm, COFs are not merely passive adsorbents but dynamic functional materials embedded within intelligent treatment architectures.

Future generations of COFs are likely to transcend static adsorption behavior through the incorporation of stimuli-responsive functionalities. Such frameworks may enable controlled, on-demand regeneration triggered by light, mild thermal inputs, or electrochemical stimuli. The rational design of these multifunctional systems, however, introduces unprecedented structural and energetic complexity. AI-guided methodologies will therefore play a critical role in navigating these expanded design spaces, balancing responsiveness, stability, and long-term durability.

Simultaneously, advances in predictive modeling and process integration point toward the rise of digital twins in water treatment infrastructure. In this framework, AI-informed models coupled to real-time operational data may enable dynamic prediction of adsorption performance, breakthrough behavior, and material lifetimes under fluctuating inlet chemistries. Such systems would transform remediation from a reactive process into a predictive, adaptive strategy, allowing treatment protocols to be adjusted with precision rather than approximation.

Yet, the realization of these capabilities depends fundamentally on data. The transition toward AI-enabled environmental engineering requires the establishment of standardized, FAIR (findable, accessible, interoperable, and reusable) data ecosystems. Critically, this includes not only high-performance materials but also negative results, failed syntheses, and performance limitations. These data sets form the statistical backbone of robust predictive frameworks, reducing bias and expanding model generalizability.

Taken together, these shifts signal a broader transformation in how remediation technologies are conceived. The central challenge is no longer solely the discovery of materials that function under controlled laboratory conditions, but the development of adaptive systems capable of learning, evolving, and operating within the complexity of real environmental matrices. In this emerging landscape, COFs, AI, and data-centric discovery frameworks converge to redefine remediation not as a static solution, but as a continuously optimized process.

Ultimately, the future of PFAS mitigation may depend less on identifying a single “perfect material,” and more on constructing intelligent discovery ecosystems capable of systematically approaching optimality.

## 7 Conclusions

The persistent challenge posed by PFAS contamination necessitates a fundamental rethinking of how porous materials designed for PFAS removal are conceived, optimized, and deployed. While covalent organic frameworks offer an unparalleled platform for molecular-level precision, their translation beyond laboratory demonstrations has been constrained by competing performance, manufacturability, end-of-use strategy, and economic requirements. In this perspective, we argue that this complexity should not be viewed as a limitation, but rather as an opportunity for methodological evolution. By repositioning COFs as selective polishing layers within hybrid treatment trains and integrating AI-guided discovery frameworks, the field can move beyond sequential trial-and-error paradigms toward systematic, multiobjective optimization strategies. Artificial intelligence, in this context, complements experimental insight by providing a navigational layer across vast chemical and operational landscapes. The convergence of data-centric workflows, physics-informed modeling, and experimental validation offers a credible pathway for accelerating the identification of COFs capable of operating within realistic water treatment infrastructures. Ultimately, the future of PFAS remediation may depend not on the discovery of a singular “ideal material,” but on the development of intelligent discovery ecosystems capable of continuously approaching optimality.

## References

[1] Buck R. C., Franklin J., Berger U., Conder J. M., Cousins I. T., De Voogt P., Jensen A. A., Kannan K., Mabury S. A., van Leeuwen S. P. (2011). Perfluoroalkyl and polyfluoroalkyl substances in the
environment: terminology, classification, and origins. Integr. Environ. Assess. Manage..

[2] Verma S., Lee T., Sahle-Demessie E., Ateia M., Nadagouda M. N. (2023). Recent
advances on PFAS degradation via thermal and nonthermal methods. Chem. Eng. J. Adv..

[3] EPA . Per- and Polyfluoroalkyl Substances (PFAS) Final PFAS National Primary Drinking Water Regulation. 2026. https://www.epa.gov/sdwa/and-polyfluoroalkyl-substances-pfas. (accessed 13 March, 2026).

[4] Burkhardt J., Speth T. F., Gorzelnik S., Gorzalski A. S., Coronell O., El-Khattabi A. R., Ateia M. (2025). How Do Novel PFAS Sorbents
Fit into Current Engineering Paradigm?. ACS
ES&T Eng..

[5] Wang Y., Darling S. B., Chen J. (2021). Selectivity
of Per- and Polyfluoroalkyl
Substance Sensors and Sorbents in Water. ACS
Appl. Mater. Interfaces.

[6] Xiao F. (2017). Emerging poly-
and perfluoroalkyl substances in the aquatic environment: A review
of current literature. Water Res..

[7] Du Z., Deng S., Bei Y., Huang Q., Wang B., Huang J., Yu G. (2014). Adsorption
behavior and mechanism
of perfluorinated compounds on various adsorbents–A review. J. Hazard. Mater..

[8] Jrad A., Das G., Alkhatib N., Prakasam T., Benyettou F., Varghese S., Gándara F., Olson M., Kirmizialtin S., Trabolsi A. (2024). Cationic covalent organic
framework for the fluorescent
sensing and cooperative adsorption of perfluorooctanoic acid. Nat. Commun..

[9] Jrad A., Garai B., Aouad S., Das G., Duncan C. M., Olson M. A., Trabolsi A. (2025). Redefining forever:
Advancements,
challenges, and opportunities in covalent organic frameworks for the
remediation of forever chemicals. Chem.

[10] Diercks C. S., Yaghi O. M. (2017). The atom, the molecule,
and the covalent organic framework. Science.

[11] Wang W., Zhou Z., Shao H., Zhou S., Yu G., Deng S. (2021). Cationic covalent organic
framework for efficient removal of PFOA
substitutes from aqueous solution. Chem. Eng.
J..

[12] Wang W., Shao H., Zhou S., Zhu D., Jiang X., Yu G., Deng S. (2021). Rapid Removal of Perfluoroalkanesulfonates
from Water
by β-Cyclodextrin Covalent Organic Frameworks. ACS Appl. Mater. Interfaces.

[13] Wang B., Lee L. S., Wei C., Fu H., Zheng S., Xu Z., Zhu D. (2016). Covalent triazine-based
framework: A promising adsorbent
for removal of perfluoroalkyl acids from aqueous solution. Environ. Pollut..

[14] Román
Santiago A., Yin S., Elbert J., Lee J., Shukla D., Su X. (2023). Imparting Selective Fluorophilic
Interactions in Redox Copolymers for the Electrochemically Mediated
Capture of Short-Chain Perfluoroalkyl Substances. J. Am. Chem. Soc..

[15] Huang J., Shi Y., Huang G.-Z., Huang S., Zheng J., Xu J., Zhu F., Ouyang G. (2022). Facile Synthesis of a Fluorinated-Squaramide Covalent
Organic Framework for the Highly Efficient and Broad-Spectrum Removal
of Per- and Polyfluoroalkyl Pollutants. Angew.
Chem., Int. Ed..

[16] Song C., Zheng J., Zhang Q., Yuan H., Yu A., Zhang W., Zhang S., Ouyang G. (2023). Multifunctionalized
Covalent Organic Frameworks for Broad-Spectrum Extraction and Ultrasensitive
Analysis of Per- and Polyfluoroalkyl Substances. Anal. Chem..

[17] Lohse M. S., Bein T. (2018). Covalent Organic Frameworks: Structures, Synthesis, and Applications. Adv. Funct. Mater..

[18] Zhang S., Wang X., He H., Yang X., Li Q., Yuan Y. (2025). Green and Large-Scale Synthesis of Covalent Organic Frameworks for
Practical Applications. Adv. Funct. Mater..

[19] Zhao W., Li H., Thuan B. D., Chen W., Zhu Q., Bahri M., Li B., Yu K., Kang C., Chen T., Wang Z., Browning N. D., Chua K. J., Zhao D. (2026). Covalent Organic Frameworks
for Water Sorption: The Importance of Framework Physical Stability. Adv. Funct. Mater..

[20] Gendy E. A., Ifthikar J., Ali J., Oyekunle D. T., Elkhlifia Z., Shahib I. I., Khodair A. I., Chen Z. (2021). Removal of heavy metals
by covalent organic frameworks (COFs): A review on its mechanism and
adsorption properties. J. Environ. Chem. Eng..

[21] Kang S., Kim Y., Kim J. (2025). Quantum Computing
Based Design of Multivariate Porous
Materials. ACS Cent. Sci..

[22] Axelrod S., Schwalbe-Koda D., Mohapatra S., Damewood J., Greenman K. P., Gómez-Bombarelli R. (2022). Learning Matter:
Materials Design
with Machine Learning and Atomistic Simulations. Acc. Mater. Res..

[23] Singh R., Uthappa U. T., Ahn Y.-H., Ray S. S. (2025). Emergence
of covalent
organic frameworks in forever chemicals Remediation: A comprehensive
and Multifaceted review. Inorg. Chem. Commun..

[24] Appleman T. D., Higgins C. P., Quiñones O., Vanderford B. J., Kolstad C., Zeigler-Holady J. C., Dickenson E. R. V. (2014). Treatment
of poly- and perfluoroalkyl substances in U.S. full-scale water treatment
systems. Water Res..

[25] EPA United States Environmental Protection Agency . Research on Per- and Polyfluoroalkyl Substances (PFAS). 2025. https://www.epa.gov/chemical-research/research-and-polyfluoroalkyl-substances-pfas (accessed 24 May, 2026).

[26] U.S. National Science Foundation . Engineering Research to Advance Solutions for Environmental PFAS (ERASE-PFAS). https://www.nsf.gov/funding/opportunities/dcl-engineering-research-advance-solutions-environmental-pfas/nsf20-090 (accessed 24 May, 2025).

[27] SERDP and ESTCP . High-Capacity Regenerable Sorbents for Treatment of PFAS. https://serdp-estcp.mil/projects/details/ae818096-e9ca-4fc0-8303-c75e58864b3b/high-capacity-regenerable-sorbents-for-treatment-of-pfas (accessed 24 May, 2026).

[28] CycloPure . DEXSORB®Welcome to the future of water treatment. https://cyclopure.com/dexsorb/ (accessed 24 May, 2026).

[29] Puraffinity . Puraffinity takes a seat at the top table in water discourse. 2024. https://www.puraffinity.com/puraffinity-takes-a-seat-at-the-top-table-in-water-discourse (accessed 25 May, 2026).

[30] Sharma V.
K., Ma X., Shetty D., Nadagouda M. N. (2025). Recent advances in covalent organic
frameworks for PFAS removal: A comparative review on 2D and 3D architectures. Chem. Eng. J..

[31] Zhao G., Zhou S., Li J., Yang S. (2026). Adsorptive removal
of per- and polyfluoroalkyl substances: From conventional porous materials
to metal-organic frameworks and covalent organic frameworks. Coord. Chem. Rev..

[32] Zhang Q., Huang Y., Dai Z., Li Y., Li Z., Lai R., Wei F., Shao F. (2025). Covalent Organic
Framework Membranes:
Synthesis Strategies and Separation Applications. ACS Appl. Mater. Interfaces.

[33] Wang H., Li Y., Xuan X., Wang K., Yao Y.-f., Pan L. (2025). Machine Learning
Accelerated Discovery of Covalent Organic Frameworks for Environmental
and Energy Applications. Environ. Sci. Technol..

[34] Butler K. T., Davies D. W., Cartwright H., Isayev O., Walsh A. (2018). Machine learning
for molecular and materials science. Nature.

[35] De
Vos J. S., Ravichandran S., Borgmans S., Vanduyfhuys L., Van Der Voort P., Rogge S. M. J., Van Speybroeck V. (2024). High-Throughput
Screening of Covalent Organic Frameworks for Carbon Capture Using
Machine Learning. Chem. Mater..

[36] Chen L., Lu Z., Chen L., Hou L., Zhang D. (2026). Chemist-Guided Human–AI
Workflow for Covalent Organic Framework Synthesis. J. Am. Chem. Soc..

[37] Wu M., Sun J., Cui Y., Fan L., Hong K., Liu W., Li Q., Lyu Z., Wang J. (2025). Active Learning-Driven Discovery
of Donor-Acceptor Covalent Triazine Frameworks for High-Performance
Photocatalysts. Adv. Funct. Mater..

[38] Ma B., Qin N., Yan Q., Zhou W., Zhang S., Wang X., Bao L., Lu X. (2026). Advancing metal organic
framework and covalent organic
framework design via the digital-intelligent paradigm. Digital Discovery.

[39] Zhang X., Hu J., Liu H., Sun T., Wang Z., Zhao Y., Zhang Y.-B., Huai P., Ma Y., Jiang S. (2025). Determining
Covalent Organic Framework Structures Using Electron Crystallography
and Computational Intelligence. J. Am. Chem.
Soc..

[40] Rajguru P., Bora P., Bhuyan C., Hazarika S. (2026). Exploring covalent
organic frameworks through the lens of computational chemistry. Mater. Adv..

[41] Jackson N. E., Savoie B. M., Statt A., Webb M. A. (2023). Introduction to
Machine Learning for Molecular Simulation. J.
Chem. Theory Comput..

[42] Cao X., Zhang Z., He Y., Xue W., Huang H., Zhong C. (2022). Machine-Learning-Aided Computational Study of Covalent Organic Frameworks
for Reversed C2H6/C2H4 Separation. Ind. Eng.
Chem. Res..

[43] Fiyadh S. S., Alardhi S. M., Al Omar M., Aljumaily M. M., Al Saadi M. A., Fayaed S. S., Ahmed S. N., Salman A. D., Abdalsalm A. H., Jabbar N. M., El-Shafi A. (2023). A comprehensive review
on modelling the adsorption process for heavy metal removal from waste
water using artificial neural network technique. Heliyon.

[44] Mallahi M. E., Akichouh E., Elyoussfi A., Salhi A., Ahari M. h., Amhamdi H., Ghalit M., Mourabit F. (2025). Artificial intelligence
in adsorption process optimization: A 2014–2024 bibliometric
analysis of research trends and developments. Results Eng..

[45] Zhang W., Huang W., Tan J., Huang D., Ma J., Wu B. (2023). Modeling, optimization
and understanding of adsorption process for
pollutant removal via machine learning: Recent progress and future
perspectives. Chemosphere.

[46] Jia X., Lynch A., Huang Y., Danielson M., Lang’at I., Milder A., Ruby A. E., Wang H., Friedler S. A., Norquist A. J., Schrier J. (2019). Anthropogenic biases
in chemical reaction data hinder exploratory inorganic synthesis. Nature.

